# NGFR Increases the Chemosensitivity of Colorectal Cancer Cells by Enhancing the Apoptotic and Autophagic Effects of 5-fluorouracil *via* the Activation of S100A9

**DOI:** 10.3389/fonc.2021.652081

**Published:** 2021-04-30

**Authors:** Hao Chen, Jintuan Huang, Chunyu Chen, Yingming Jiang, Xingzhi Feng, Yi Liao, Zuli Yang

**Affiliations:** ^1^ Department of Gastrointestinal Surgery, The Sixth Affiliated Hospital of Sun Yat-sen University, Guangzhou, China; ^2^ Guangdong Institute of Gastroenterology, The Sixth Affiliated Hospital of Sun Yat-sen University, Guangzhou, China

**Keywords:** colorectal cancer, NGFR, S100A9, apoptosis, autophagy, chemosensitivity

## Abstract

Colorectal cancer (CRC) is currently the third leading cause of cancer-related deaths worldwide, and 5-fluorouracil (5-FU)-based chemotherapies serve as important adjuvant therapies before and after surgery for CRC. However, the efficacy of CRC chemotherapy is limited by chemoresistance, and therefore the discovery of novel markers to indicate chemosensitivity is essential. Nerve growth factor receptor (NGFR), a cell surface receptor, is involved in cell death and survival. Our previous study indicated that NGFR acts as a tumor suppressor, and high expression is associated with better outcomes in patients receiving 5-FU-based adjuvant chemotherapy after surgery. The aim of this study was to investigate the effect of NGFR on the chemotherapeutic response in CRC. Chemosensitivity was investigated using DLD1 and HCT8 cells after NGFR transfection. Apoptosis was investigated by flow cytometry. Autophagy was assessed using GFP-LC3B transient transfection. Gene expression was measured using an mRNA microarray. Beclin-1 and Bcl-2 protein expressions were assessed by western blot. NGFR and S100 calcium-binding protein A9 (S100A9) expressions in CRC patients were investigated by immunohistochemistry. The results showed that the half maximal inhibitory concentration of NGFR-transfected cells was lower than that of controls in DLD1 and HCT8 cells after 5-FU treatment, and cell viability was lower than in empty-vector cells. Tumor sizes were also smaller than in empty-vector cells *in vivo*. The percentages of apoptotic and autophagic cells were higher in NGFR-transfected cells. NGFR elevated the expression of S100A9 after 5-FU treatment. The combination of Bcl-2 and Beclin-1 was significantly suppressed by overexpressed NGFR. Five-year overall and disease-free survival in NGFR+/S100A9+ patients was better than in NGFR−/S100A9− patients. This study’s findings suggest that NGFR may serve as a marker predicting CRC patients’ chemosensitivity.

## Introduction

Colorectal cancer (CRC) is currently the third leading cause of cancer-related deaths in the world (1). Chemotherapies serve as important adjuvant therapies for CRC before and after surgery. Among them, 5-fluorouracil (5-FU), alone or combined with other agents, is the most widely used chemotherapeutic drug for advanced CRC patients (2). Even though adjuvant 5-FU-based chemotherapeutic treatment has led to an increase in survival rates, over 90% of CRC patients still suffer from treatment failure due to therapeutic resistance (3). The mechanisms of resistance to 5-FU are poorly understood. Therefore, elucidating chemoresistance and finding a biomarker to indicate chemosensitivity would be helpful for improving the efficiency of chemotherapy in patients with 5-FU-resistant CRC.

Nerve growth factor receptor (NGFR), also known as p75 neurotrophin receptor and CD271, belongs to the tumor necrosis factor receptor (TNFR) superfamily. As a cell surface receptor, NGFR is involved in various signal transduction pathways, depending on the cell type and cell differentiation status (4, 5). Due to its multiple involvement, NGFR induces different cellular responses. NGFR overexpression promoting tumor migration and invasion has been observed in some metastatic cancers (6–8). On the other hand, NGFR has also been reported to suppress tumor growth and/or metastasis in prostate and bladder cancers (9–11). Previously, we reported that NGFR plays a role as a tumor suppressor gene, and it could be silenced by epigenetic modification. NGFR expression in CRC cells inhibits cell proliferation and invasion through the induction of G1 phase arrest and cell apoptosis. As a candidate tumor suppressor, NGFR has independent prognostic potential in CRC (12). By further analyzing immunohistochemistry (IHC) data, we found that, compared to patients with low NGFR expression, patients with high expression have better outcomes after 5-FU-based chemotherapy following surgery. However, the role of NGFR in 5-FU-based chemotherapy for CRC remains unknown. For this reason, we performed a gene microarray analysis, which indicated that NGFR might mediate 5-FU chemosensitivity of CRC through S100 calcium-binding protein A9 (S100A9).

Protein S100A9, also known as calgranulin B, MRP14, and calprotectin, is a member of the S100 subfamily of cytoplasmic EF-hand Ca2+-binding proteins. S100A9 usually works with S100A8 as a protein complex released due to apoptotic or cytotoxic effects, such as those of chemotherapeutic agents against various tumor cells (13, 14). It has been established that membrane-associated intracellular S100A9 and soluble extracellular S100A9 have different cellular functions. Intracellular S100A9 might be involved in phagocyte nicotinamide adenine dinucleotide phosphate oxidase activation (15). The extracellularly secreted form exerts cell growth–promoting activities at low concentrations (16), while it induces cell death at higher concentrations (14). However, the receptor upon which the apoptotic-inducing property relies is unknown (14, 16).

The Bcl-2 family, including Bcl-2, Bcl-xL and Mcl-1, are well-defined anti-apoptotic mediators. However, the roles of Bcl-2 in inhibiting autophagy are becoming more interesting. The Bcl-2 proteins exert a cytoprotective function to antagonize Bax and Bak to prevent apoptosis. The Bcl-2 homology 3 (BH3) domain only protein, Beclin 1 (17), is an essential autophagy initiator. Beclin 1 recruits key autophagic proteins to forming a pre-autophagosomal structure, the core complex consisting Beclin 1, Vps34, and Vps15 (18). Therefore, Beclin 1 is a key determining factor as to whether cells undergo autophagy or apoptosis (19). So, the interaction and expression levels of Beclin 1 and Bcl-2 are key determinants as to whether cells are resistant to apoptosis or autophagy during tumorigenesis and chemotherapy.

This study investigated whether NGFR enhances 5-FU-induced apoptosis and autophagy by activating S100A9 in CRC.

## Materials and Methods

### Cell Lines and Cell Culture

Human CRC cell lines DLD1 and HCT8 were obtained from the American Type Culture Collection (Manassas, VA, USA) and were maintained in an RPMI 1640 medium (Thermo Fisher Scientific, Waltham, MA, USA) with 10% fetal bovine serum (Corning Cellgro Inc., Herndon, VA, USA). The cells were cultured at 37°C in a 5% CO_2_ incubator.

### Patients and Samples

For Tissue microarrays (TMA) construction, 251 paraffin-embedded samples of primary colorectal adenocarcinomas patients were obtained, who underwent surgery and were then treated with 5-FU based chemotherapy (mFOLFOX6 or XELOX) at the Sixth Affiliated Hospital of Sun Yat-sen University (SYSU) between 2009 and 2012. Patient enrollment criteria included: pathological confirmation of stage II/III CRC patients, the undergoing of curative surgical resection, absence of preoperative chemotherapy or radiotherapy, availability of tumor specimen, and complete follow-up information. The median follow-up time was 1460 days. A written informed consent from each patient regarding tissue sampling had been obtained and the study were reviewed and approved by the Medical Ethics Committee of the Sixth Affiliated Hospital, Sun Yat-sen University. Baseline information and clinicopathological features of the patients were listed in **Supplementary Table 1**.

### NGFR-Expressing Plasmid and Transfection

The CRC lines DLD1 and HCT8 were transfected with a pLVX-IRES-puro-NGFR plasmid or a pLVX-IRES-puro-Mock empty vector (OriGene, Rockville, MD, USA) using a Lipofectamine 3000 transfection reagent (Invitrogen, Carlsbad, CA, USA). Stable NGFR- and vector-expressing clones were selected for further study.

### Colony Formation Assays

For colony formation assays with a monolayer culture, DLD1 and HCT8 cells stably transfected with an NGFR expression plasmid or an empty vector (1 × 10 ^3^/well) were plated in a six-well plate and treated with 5-FU for 2 h (200 *μ*m/L for DLD1; 100 *μ*m/L for HCT8). After treating with puromycin (2.5 *μ*g/L) for 14–21 days, the surviving colonies (≥50 cells/colony) were counted after Giemsa staining.

### Cell Viability Assay

For cell viability measurements, 5-FU (Sigma-Aldrich, St. Louis, MO, USA) was dissolved in dimethyl sulfoxide (DMSO) with a stock concentration of 100 mM and freshly diluted to the desired concentrations with a culture medium. The final DMSO concentration was 0.05% (v/v). An MTT assay was performed to examine the effect of 5-FU on the viability of transfected DLD1 and HCT8 cells treated with different concentrations of 5-FU (1–256 *μ*M). The protocol was described in our previous study (12). Absorbance at a wavelength of 490 nm was measured with a Varioskan Flash (Thermo Fisher, Waltham, MA, USA). The half maximal inhibitory concentrations (IC50) were analyzed using Prism 6.0 (GraphPad Software, Inc., La Jolla, CA, USA).

### Apoptosis Analysis

In total, 5 × 10^5^ transfected DLD1 and HCT8 cells were seeded into 10-cm^2^ six-well plates, treated with 5-FU, and collected by centrifugation. The pellet cells were stained using an Annexin V-FITC detection kit (Multi Sciences [Lianke] Biotech, Hangzhou, China) and analyzed using a FACS-Canto TMII flow cytometer (BD Biosciences, San Jose, CA, USA).

### Cell Cycle Assay

HCT8 cells stably transfected with P75NGFR or empty vector, after 5-FU treatment for 48 hours, were fixed in 70% ethanol and stained with 50 mg/mL propidium iodide (BD Pharmingen). The cells were then sorted by FACS-Canto TMII flow cytometer (BD Biosciences, San Jose, CA, USA), and the cell-cycle profiles were analyzed using the Flowjo V10 software.

### Autophagy Detection

Autophagosome formation, one of the features of autophagy, can be detected by endogenous LC3 or GFP-LC3 puncta incorporated into autophagic vacuoles. Sterile 12-mm cover slips were seeded with 5 × 10^5^ transfected DLD1 and HCT8 cells into six-well plates and then treated with 5-FU. After attachment overnight, the cells were washed with phosphate buffered saline (PBS) twice, and the medium was replaced with a serum-free medium for 24 h. Then, the cells were transiently transfected with a GFP-LC3B-expressing vector (OriGene, Rockville, MD, USA). After treatment for 32 h, the cells were washed with cold PBS and then fixed with 4% formaldehyde in PBS (pH 7.4) for 20 min at room temperature. Cells presenting a mostly diffuse distribution of GFP-LC3B in the cytoplasm and nucleus were considered non-autophagic, whereas cells representing several intense punctate GFP-LC3B aggregates with no nuclear GFP-LC3B were classified as autophagic.

### Confocal Microscopy

Transfected cells (5 × 10^5^) were seeded in six-well plates preloaded with sterilized glass cover slips. After 5-FU treatment, cells on the cover slips were washed twice with PBS and fixed with 4% paraformaldehyde for 30 min at room temperature. After washing with PBS three times, the cover glasses were carefully mounted onto microscope glasses containing a drop of ProLong Gold Antifade reagent with DAPI (Invitrogen, Carlsbad, CA, USA). Finally, the slides were sealed and analyzed using a confocal microscope (Leica Microsystems, Wetzlar, Germany).

### Immunohistochemistry

A ChemMate EnVision Detection Kit (Dako, Carpinteria, CA, USA) was used. The TMA sections were incubated with an NGFR antibody (P08138, Abgent, San Diego, CA, USA) and an S100A9 antibody (ab92507, Abcam, Cambridge, UK) at 4°C overnight. The IHC protocol was described in our previous study (12). The protein expressions were scored on a four-point scale based on the intensity of cytoplasmic staining as follows: 0, negative; 1, weak; 2, moderate; and 3, strong. According to staining scores, patients were divided into two groups: low (−) expression (scores 0 and 1) and high (+) expression (scores 2 and 3). Immunostaining was scored independently on separate occasions by two investigators who were blinded to the patients’ clinical information.

### Western Blot Analysis

Cells were harvested from culture dishes and lysed in a lysis buffer. The western blot analysis protocol was described in our previous study (12). The antibodies used were cleaved caspase-3, Bcl-2, LC3B, Thymidylate Synthase (TYMS), UMP-CMP kinase (Cell Signaling Technology, Danvers, MA, USA), and glyceraldehyde 3-phosphate dehydrogenase (GAPDH; Santa Cruz, CA, USA). Detection was performed using an ECL kit (Pierce Chemical, Dallas, TX, USA), and the blots were developed using a ChemiDoc Touch imaging system (Bio-Rad Laboratories, Hercules, CA, USA).

### *In Vivo* Subcutaneous Tumor Model

Ten male BALB/c nude mice (6-8weeks old) were obtained from Laboratory Animal Center of SYSU. Mice were randomly divided into two groups: group 1, mice injected with HCT8-NGFR cells and treated with 5-FU; and group 2, mice injected with HCT8-PURO cells and treated with 5-FU. Transfected HCT8 cells (5 × 10^6^ cells in 0.2 ml of PBS) were injected subcutaneously into the right dorsal flank of each mouse (five mice/group). After subcutaneously inoculating tumor cells in the left right dorsal flank of the mice, the animals were treated with 5-FU (5 mg/kg/day) by intraperitoneal injection for 10 consecutive days. Tumor volumes were measured every three days for three to four weeks using the following formula: short diameter (2) × long diameter/2. All *in vivo* experimental protocols were approved by the Animal Care Committee of Sun Yat-sen University.

### RNA Preparation and Microarray Analysis

Gene microarray analysis was performed by Phalanx Biotech Group (Hsinchu, Taiwan) and included RNA amplification, labeling of probes, hybridization, and data extraction. Briefly, total RNA was extracted from DLD1-PURO cells after three time individual 5-FU treatment as a control group (PURO_H001, PURO_H002, PURO_H003) and DLD1-NGFR cells after three time individual 5-FU treatment as an experimental group (P75_H004, P75_H005, P75_H006) using a Trizol reagent (TaKaRa Bio Inc., Mountain View, CA, USA) according to the manufacturer’s instructions. Gene expression profiling was performed using a Human OneArray v6.1 microarray (Phalanx Biotech Group, Hsinchu, Taiwan) containing 31,741 human genome probes and 938 experimental control probes. After hybridization, the arrays were washed and scanned, and then gene expression results (**Figures S1** and **S2**) were extracted using a DNA Microarray Scanner G2565B (Agilent Technologies, Santa Clara, CA, USA) according to the manufacturer’s instructions. Raw fluorescence intensity values (**Figure S3**) were normalized and log-transformed using Cluster 3.0 (University of Tokyo, Japan).

### Quantitative Real-Time Polymerase Chain Reaction (qRT-PCR)

Total RNAs from frozen tissue samples or CRC cell lines were extracted using TRIZOL reagent (Invitrogen) following the manufacturer’s protocol (20). The qRT-PCR protocol was described in our previous study (12). GAPDH was used as an internal control to normalize messenger RNA (mRNA) levels, and fold change of expression was calculated according to the ΔΔCt method (21). All experiments were conducted in triplicate. The primer sequences are listed in **Supplementary Table 2**.

### Immunoprecipitation

Co-immunoprecipitation (co-IP) experiments were conducted to reveal the interactions between endogenous Bcl-2 and endogenous Beclin-1 in the presence and absence of NGFR. DLD1 and SW480 cells were subcultured in a six-well plate. NGFR overexpression and empty plasmids, as well as NGFR short hairpin RNA (shRNA) and control plasmids, were transfected into CRC cells using a Lipofectamine 3000 reagent (Invitrogen, Carlsbad CA, USA) according to the manufacturer’s protocol. A 5-FU concentration of 100 *μ*M was applied to transfected cells for 12 h. After treatment, cells were lysed in an IP lysis buffer. Protein concentrations were measured using a Bradford protein assay (Bio-Rad Laboratories, Hercules, CA, USA). Equal amounts of proteins were incubated with Bcl-2 antibodies at 4°C overnight and then incubated with Dynabeads Protein G (Invitrogen, Carlsbad, CA, USA) for 1 h. The beads were washed in an IP lysis buffer five times and then eluted and analyzed using western blot to detect the presence of endogenous Beclin-1. Reversely, the same amounts of proteins were incubated with Beclin-1 antibodies and then analyzed using western blot to detect the presence of endogenous Bcl-2 following the same procedure.

### Statistical Analysis

All of the experiments were repeated at least three times. Statistical analyses were performed using the SPSS 22.0 software (SPSS Inc., Chicago, IL, USA) for Windows. Statistical analyses for cell line experiments were performed by Student’s t-test or Mann–Whitney U-test. For *in vivo* assays in xenografts, statistical analyses were performed by two-way ANOVA. A P-value<0.05 was considered statistically significant (*P < 0.05, **P < 0.01, ***P < 0.001).

## Results

### NGFR Increases the Chemosensitivity of CRC Cells to 5-FU

**Figure 1:**
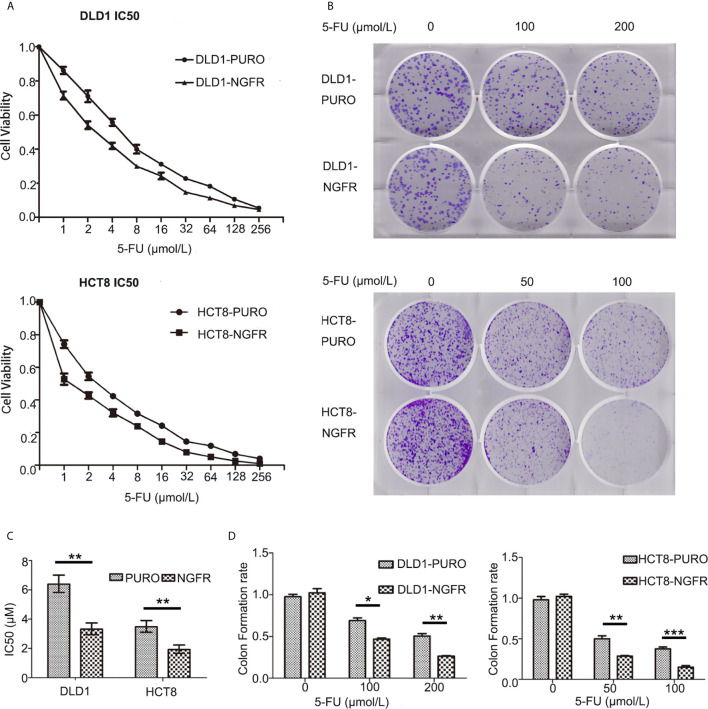
NGFR contributed to colorectal cancer cell suppression after 5-FU treatment *in vitro.* **(A)** An MTS assay was performed to investigate the viability of overexpressed NGFR–transfected DLD1 and HCT8 cells at different 5-FU concentrations (0–256 *µ*mol/L). **(B)** A colony formation assay was performed using overexpressed NGFR–transfected DLD1 and HCT8 cells. **(C)** The IC50 values were determined as indicated on the plot (t-test; P < 0.001 for DLD1 cells, P < 0.01 for HCT8 cells). **(D)** Colony formation assays indicated that NGFR inhibited overexpressed NGFR–transfected DLD1 and HCT8 cell colony formation (cell viability) with increasing 5-FU concentrations (0–200 *µ*M for DLD1 cells, 0–100 *µ*M for HCT8 cells; t-test; P < 0.01 for DLD1 cells, P < 0.001 for HCT8 cells). *P < 0.05; **P < 0.01; ***P < 0.001.

**Figure 2:**
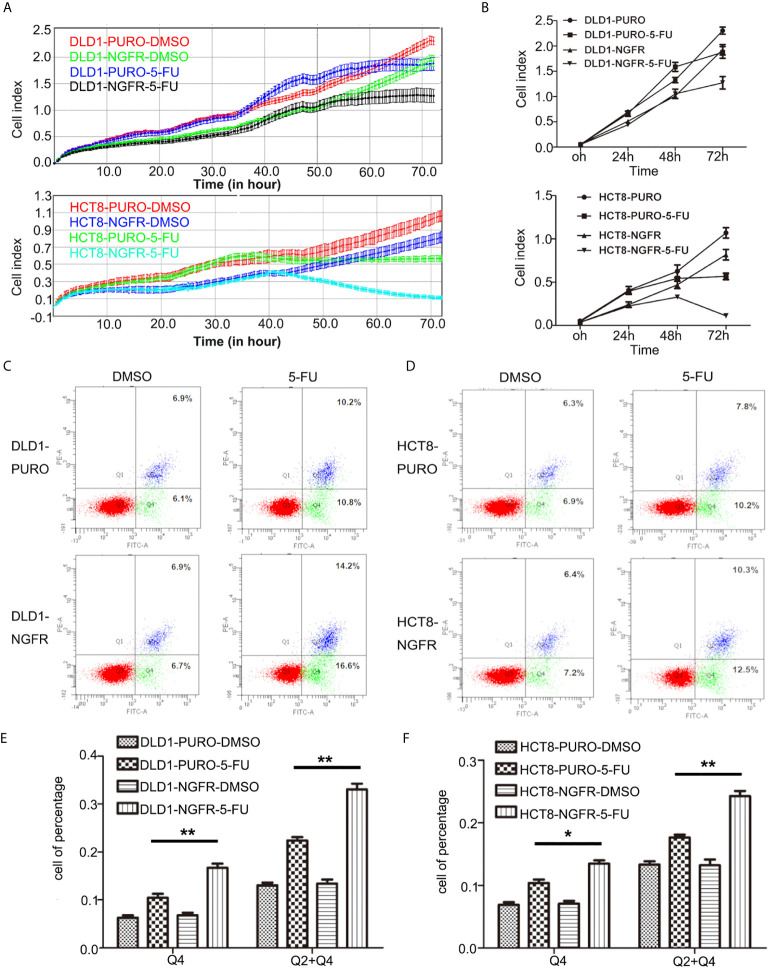
NGFR increases the chemosensitivity of CRC cells to 5-FU by elevating 5-FU-induced apoptosis. **(A, B)** Real-time cellular analysis confirmed the results of the colony formation assays regarding the inhibition of overexpressed NGFR–transfected DLD1 and HCT8 cell growth with 5-FU treatment (t-test; P < 0.01 for DLD1 cells). Overexpressed NGFR–transfected **(C)** DLD1 and **(D)** HCT8 cells treated with 5-FU for 48 h. Cell apoptosis was quantitated on the graph using an Annexin V:PE apoptosis detection kit and a flow cytometer. The apoptosis rate was significantly increased in overexpressed NGFR–transfected **(E)** DLD1 and **(F)** HCT8 cells after 5-FU treatment (Q4, early apoptosis; Q2 + Q4, total apoptosis; t-test; P < 0.01 for DLD1 cells, P < 0.01 for HCT8 total apoptosis cells, P < 0.05 for HCT8 early apoptosis cells). *P < 0.05; **P < 0.01.

First, we evaluated the effect of NGFR on the chemosensitivity of CRC cells to 5-FU. The IC50 of NGFR-transfected cells was significantly lower than that of controls in both DLD1 (IC50^vector^ vs. IC50^NGFR^: 6.388 [95%CI: 5.824, 7.007] vs. 3.316 [[95%CI: 2.945, 3.374] *μ*mol/L, P < 0.01) and HCT8 cells (IC50^vector^ vs. IC50^NGFR^: 3.479 [3.104, 3.900] vs. 1.926 [1.633, 2.231] *μ*mol/L, P < 0.01) after treatment with different 5-FU concentrations (**Figures 1A, C**). To confirm the effect of NGFR on chemosensitivity, colony formation was performed to assess CRC cell growth after treatment with 5-FU. The results showed that the growth of overexpressed NGFR–transfected CRC cells was significantly inhibited compared to empty vector–transfected DLD1 (vector vs. NGFR: 0.506 ± 0.028 vs. 0.263 ± 0.008, P < 0.01) and HCT8 cells (vector vs. NGFR: 0.376 ± 0.040 vs. 0.150 ± 0.033, P < 0.001; **Figures 1B, D**). To confirm the IC50 results, we performed real-time cellular analysis (RTCA) to investigate the cell viability of DLD1 and HCT8 cells stably transfected with the NGFR expression plasmid and the empty vector after treatment with 5-FU. The results showed that after treatment with 5-FU, overexpressed NGFR–transfected cells exhibited lower viability than empty vector–transfected DLD1 (vector vs. NGFR: 0.813 ± 0.056 vs. 0.661 ± 0.073, P < 0.01) and HCT8 cells (vector vs. NGFR: 0.530 ± 0.046 vs. 0.137 ± 0.025, P < 0.001; **Figures 2A, B**). Taken together, the results indicated that NGFR increased the chemosensitivity of CRC cells to 5-FU.

### NGFR Enhances 5-FU-Induced Apoptosis and Autophagy of CRC Cells

**Figure 3:**
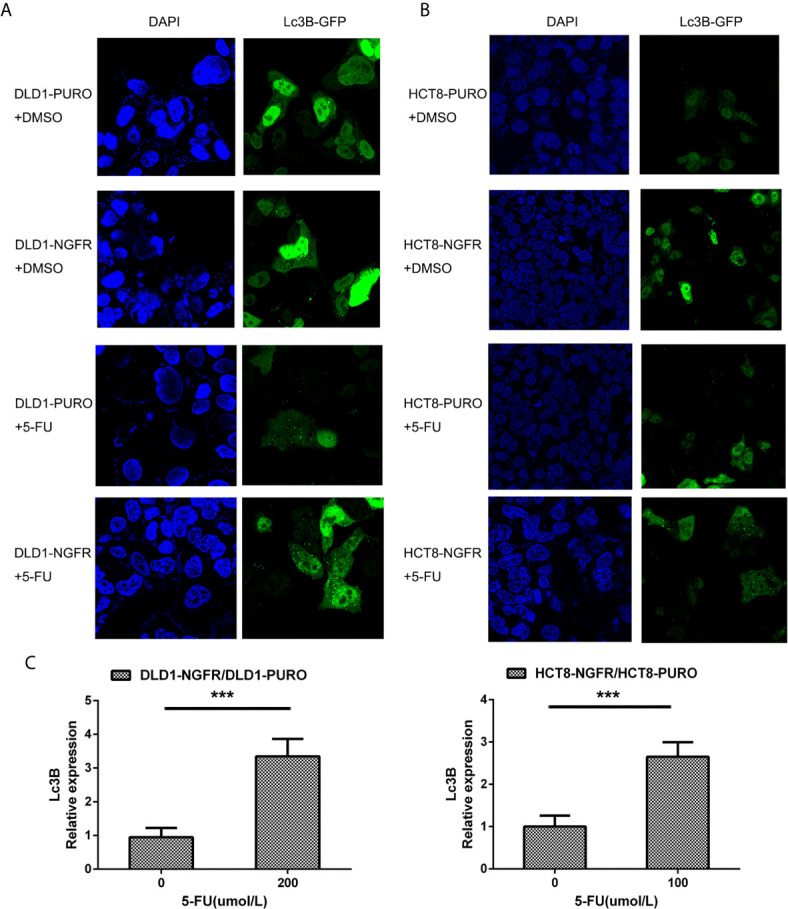
NGFR promoted 5-FU-induced autophagy of CRC cells. Overexpressed NGFR–transfected DLD1 and HCT8 cells were treated with 5-FU for 48 h. The cellular autophagy induced by **(A)** 200 *µ*M of 5-FU for DLD1 and **(B)** 100 *µ*M for HCT8 cells was examined by confocal microscopy with transfection with a GFP-LC3B vector. **(C)** Autophagosome-expressing LC3B (green dot) was quantitated in overexpressed NGFR–transfected compared to empty vector–transfected DLD1 and HCT8 cells treated with 5-FU (t-test; ***P < 0.001).

**Figure 4:**
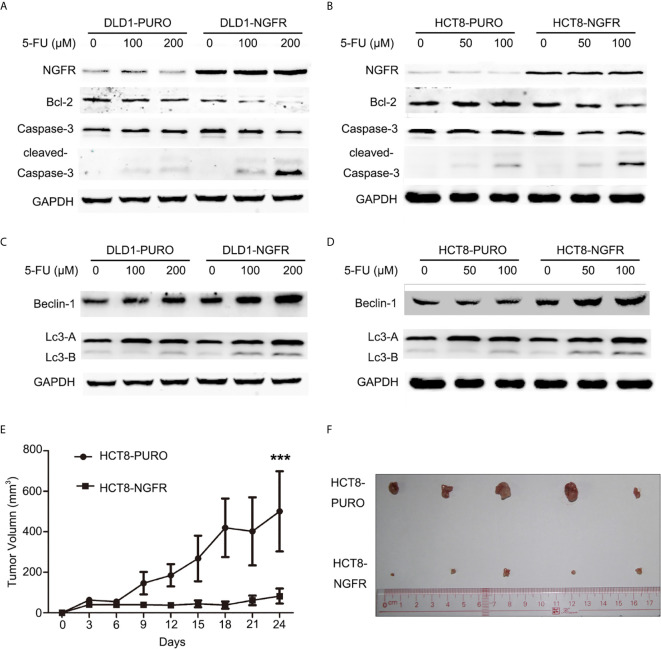
NGFR overexpression enhanced 5-FU-induced apoptosis and autophagy of CRC cells. Apoptosis markers (Bcl-2, caspase-3, and cleaved caspase-3) were detected in **(A)** DLD1 and **(B)** HCT8 cells treated with 5-FU (0, 100, and 200 *µ*M for DLD1 cells; 0, 50, and 100 *µ*M for HCT8 cells) for 48 h by western blot. Autophagy markers (Beclin-1, LC3A [upper band], and LC3B [lower band]) were detected in **(C)** DLD1 and **(D)** HCT8 cells treated with 5-FU (0, 100, and 200 *µ*M for DLD1 cells; 0, 50, and 100 *µ*M for HCT8 cells) for 48 h by western blot. The role of NGFR in tumor suppression of CRC with 5-FU-based therapy was investigated *in vivo*. The tumor **(E)** volume and **(F)** size of a xenograft mouse model in overexpressed NGFR–transfected HCT8 cells were smaller than those with empty vector–transfected HCT8 cells after 5-FU treatment (t-test; ***P < 0.001).

To determine whether tumor cell growth inhibition by NGFR was related to cell cycle arrest and apoptosis, we used flow cytometry. There were no significant differences in cell cycle phase distribution between the NGFR-transfected and control groups after treatment with 5-FU. We further evaluated the effect of NGFR expression on apoptosis after treatment with 5-FU. Flow cytometry (**Figures 2C, D**) showed that the percentage of apoptotic cells was significantly higher in NGFR-transfected cells than in controls in both early apoptosis of DLD1 (vector vs. NGFR: 10.4% ± 1.4% vs. 16.7% ± 1.5%, P < 0.01; **Figure 2E** left) and HCT8 cells (vector vs. NGFR: 10.1% ± 0.3% vs. 13.5% ± 0.9%, P < 0.05; **Figure 2F** left) and total apoptosis of DLD1 (vector vs. NGFR: 21.3% ± 1.4% vs. 32.8% ± 1.5%, P < 0.01; **Figure 2E** right) and HCT8 cells (vector vs. NGFR: 17.1% ± 0.8% vs. 24.3% ± 1.4%, P < 0.01; **Figure 2F** right). The effect of NGFR on autophagy was also explored. Cells with NGFR overexpression or an empty plasmid were transiently transfected with GFP-LC3B plasmids. When autophagy was induced, punctate fluorescence became visible. As shown in **Figures 3A, B**, obvious dot-like fluorescence was observed in NGFR-transfected cells but not in control cells. All the cells, including floating cells, were harvested, and after 24h of serum starvation, autophagy was assessed by western blot. The percentage of autophagic cells was significantly higher in NGFR-transfected cells than in controls (3.460 ± 0.517 times in DLD1, P < 0.001 and 3.710 ± 0.345 times in HCT8, P < 0.001; **Figure 3C**). To confirm our results of *in vitro* experiments, protein expression of apoptosis and autophagy markers were evaluated using Western-blot. Western-blot experiment showed an increased cleaved caspase-3 expression and decreased antiapoptotic protein Bcl-2 expression (**Figures 4A, B**), along with the increased autophagic protein Beclin-1 and autophagy marker protein LC3B expression (**Figures 4C, D**), when NGFR overexpression CRC cells treated with 5-FU. These results indicated that NGFR enhanced 5-FU-induced apoptosis and autophagy of CRC cells.

### NGFR Inhibits Tumor Growth in Nude Mice After 5-FU Treatment

We evaluated the effects of NGFR on tumor growth of CRC in mouse tumor models of NGFR- and vector-transfected HCT8 stable cell clones. The tumor sizes of NGFR transfectants were significantly smaller than those of vector transfectants after an intraperitoneal injection of 5-FU (vector vs. NGFR: 500.9 ± 198.0 mm^3^ vs. 82.6 ± 36.8 mm^3^, P < 0.001) (**Figures 4E, F**), confirming that NGFR acts as a chemotherapeutic enhancer in CRC.

### NGFR Activates S100A9 After 5-FU Treatment

**Figure 5:**
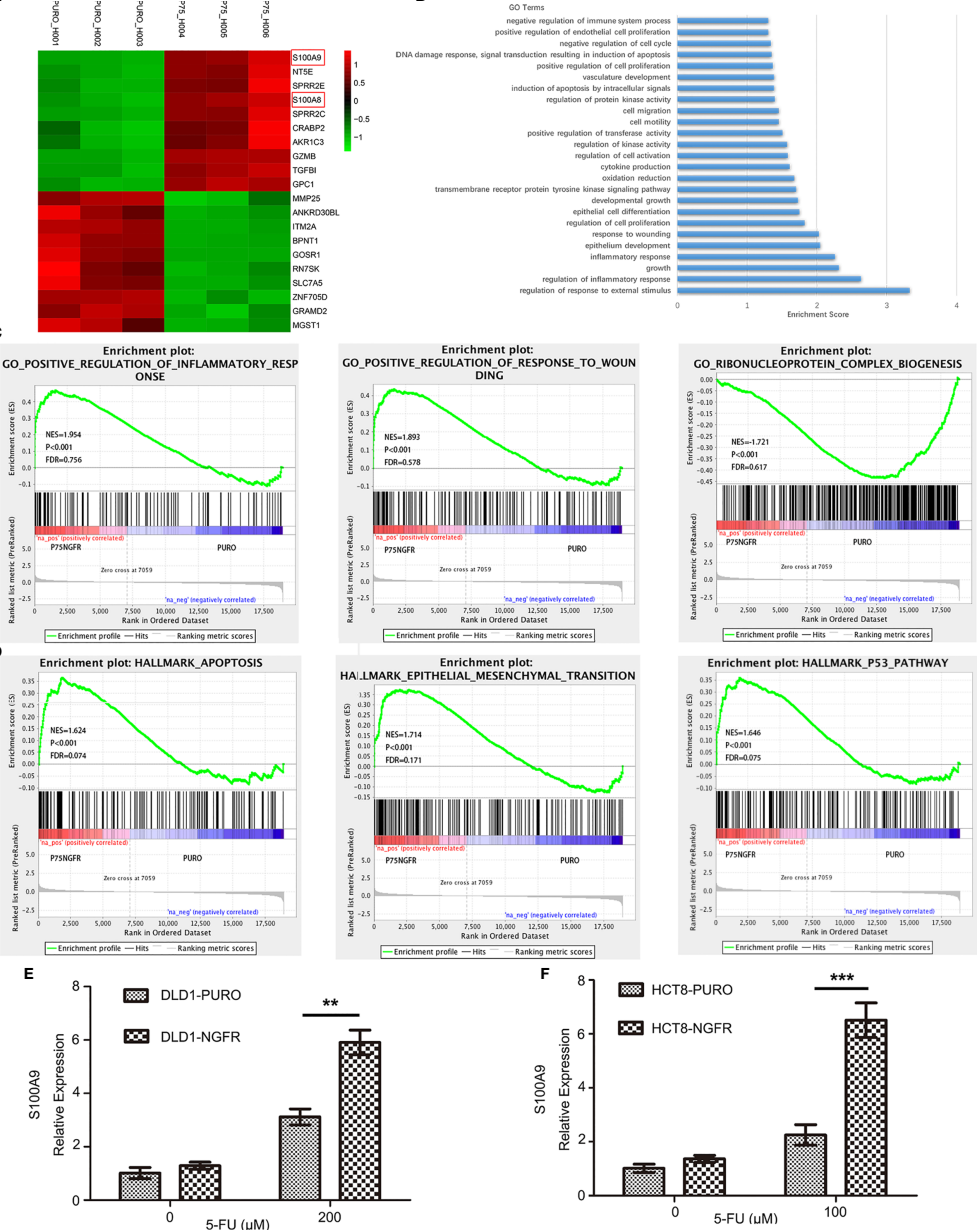
RNA sequencing revealed potential downstream NGFR signaling. **(A)** The top 10 upregulated genes (red) and top 10 downregulated genes (green). S100A8 and S100A9 are marked with red frame. **(B, C)** Gene Ontology analysis of altered genes involved in biological process functions. **(D)** Gene Set Enrichment Analysis revealed enrichment of NGFR-associated genes. S100A9 upregulation shown in graphs of overexpressed NGFR–transfected **(E)** DLD1 and **(F)** HCT8 cells after 5-FU treatment. t-test; **P < 0.01; ***P < 0.001.

To identify the cell signaling pathway in which NGFR magnified the apoptotic and autophagic effects of 5-FU, an mRNA expression microarray was performed. The microarray validated the mRNA expression changes of 31,741 genes caused by NGFR overexpression. In total, 118 genes were markedly downregulated, whereas 126 were considerably upregulated. The histogram in **Supplementary Figure 1** shows the fold change between the experimental and control groups (NGFR vs. PURO). The volcano plot in **Supplementary Figure 2** shows the distribution of differentially expressed genes between the experimental and control groups. The hierarchical clustering tree in **Supplementary Figure 3** shows the gene expression pattern similarities of the 244 genes in the experimental and control groups. The top 10 upregulated and top 10 downregulated genes are listed in **Figure 5A**.

Gene Ontology (GO) analysis was performed to identify related biological process functions of the identified genes (**Figure 5B**). Consistent with our *in vitro* and *in vivo* observations, the GO results also showed that the NGFR-regulated genes were mostly involved in pathways such as cell proliferation, cell growth, apoptosis, epithelial cell differentiation, response to wounding, and inflammatory response (**Figure 5C**). As the GO analysis only included the significant genes, we further confirmed the potential pathways by the Gene Set Enrichment Analysis (GSEA) pre-ranked tool of all genes. The results showed that enrichment of NGFR was associated with genes in hallmark apoptosis, hallmark epithelial mesenchymal transition, and the hallmark p53 pathway (**Figure 5D**).

The altered genes were selectively confirmed by qRT-PCR, which revealed similar altered gene expressions in NGFR-transfected cells. Among the upregulated genes, S100 family members exhibited the greatest fold change and thus attracted our attention. S100A9 was the most upregulated gene after 5-FU treatment (4.407 ± 0.256 times more upregulated than in control cells; **Figure 5A** and **Supplementary Figure 3**). S100A4 and S100A8 were also considerably upregulated (1.536 ± 0.090 and 2.142 ± 0.108 times, respectively, than in control cells; **Figure 5A** and **Supplementary Figure 3**), suggesting that S100 family members might play an important role in NGFR-induced enhancement of the apoptotic and autophagic effects of 5-FU. To confirm the microarray data, we performed qPCR to determine the effect of NGFR on S100A9 mRNA expression. The results showed that NGFR significantly elevated the expression of S100A9 mRNA after 5-FU treatment (1.897 ± 0.135 times compared to controls in DLD1, P < 0.01, **Figure 5E**; 2.889 ± 0.322 times in HCT8, P < 0.001; **Figure 5F**).

### NGFR-S100A9 Co-Expression Improved the Outcomes of CRC Patients After 5-FU Treatment

**Figure 6:**
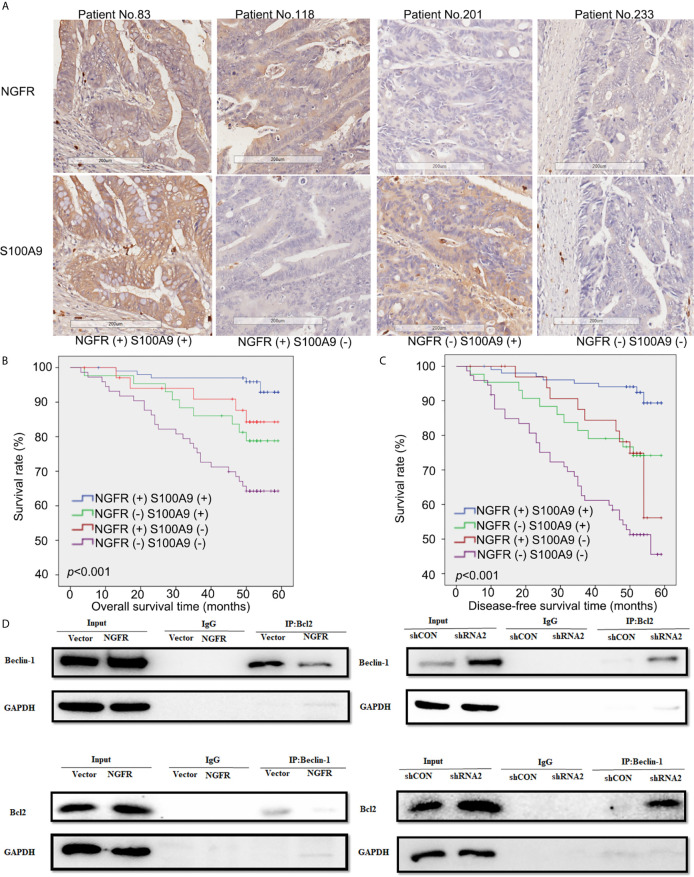
Low expressions of NGFR and S100A9 were associated with poor prognosis in patients with CRC after 5-FU-based chemotherapy. **(A)** Samples of CRC patients stained by immunohistochemistry. The expressions of NGFR and S100A9 are shown in the cytoplasm (brown stain) of CRC cells. A Kaplan-Meier plot showed that NGFR and S100A9 low co-expression was associated with poor **(B)** overall survival and **(C)** disease-free survival in patients with CRC after 5-FU-based chemotherapy. **(D)** DLD1 and SW480 cells were transfected with an NGFR overexpression plasmid and an shRNA plasmid following a 48-h 5-FU treatment. Cell lysates were then extracted for immunoprecipitation with anti-Beclin-1 or anti-Bcl-2 antibodies, and precipitates were immunoblotted with anti-Bcl-2 or anti-Beclin-1 antibodies. The upper band shows that the combination of Bcl-2 and Beclin-1 was considerably suppressed by overexpressed NGFR. The lower band shows that when NGFR was downregulated, the combination of Bcl-2 and Beclin-1 was markedly elevated.

To evaluate the effect of NGFR and its potential target S100A9 on the outcomes of CRC patients after 5-FU treatment, we recruited 251 stage II/III CRC patients receiving the same standard 5-FU-based chemotherapy after surgery. NGFR and S100A9 IHC analyses were performed (**Figure 6A**), The protein expressions of NGFR and S100A9 revealed strong positive correlations between NGFR and S100A9 protein expressions (40.2% NGFR+/S100A9+ [101/251]; 17.1% NGFR−/S100A9+ [43/251]; 13.9% NGFR+/S100A9− [35/251]; 28.7% NGFR−/S100A9− [72/251]; P < 0.001; **Table 1**). These results indicated that S100A9 was activated by NGFR after 5-FU treatment in CRC cells and played an important role in chemosensitivity. However, no significant association between clinicopathological features and NGFR expression level was observed, as well as S100A9 expression level (**Supplementary Table 1**). Kaplan–Meier analysis showed that the five-year overall survival of NGFR+/S100A9+ patients was better than that of NGFR−/S100A9+ patients, and that of NGFR+/S100A9− patients was significantly better than that of NGFR−/S100A9− patients (57.541 ± 0.709, 54.928 ± 1.940, 53.321 ± 1.946, and 47.947 ± 1.991 months, respectively, P < 0.001; **Figure 6B**). These results, as well as those of five-year disease-free survival (56.673 ± 0.871, 52.209 ± 2.113, 50.980 ± 2.357, and 42.897 ± 2.242 months, respectively, P < 0.001; **Figure 6C**), indicated that NGFR and S100A9 combined can improve the outcomes of CRC patients after 5-FU treatment. Multivariate Cox regression analysis for all 251 patients showed that NGFR high expression, S100A9 high expression and high differential grade are independent protect factors for both OS and DFS. Older age and pN positive are independent risk factors for OS and DFS, individually. Also, Tumor located in rectum has high risk than tumor located in colon for DFS (**Table 2**).

**Table 1:** Correlation between NGFR and S100A9 protein expression.

NGFR Expression	S100A9 expression		p value
	S100A9 (+)	S100A9 (-)	
NGFR (+)	101 (40.2%)	35 (13.9%)	*p*<0.001
NGFR (-)	43 (17.1%)	72 (28.7%)

(+): high expression; (-): low expression.

**Table 2:** Multivariate analysis of disease-free survival and overall survival in 251 CRC patients.

Factors	N	DFS		OS	
		HR (95% CI)	P-value	HR (95% CI)	P-value
Age, years		1.236(0.754,2.027)	0.400	1.876(1.008,3.492)	0.047
<60	142				
≥60	109				
Sex		1.185(0.715,1.962)	0.511	0.921(0.509,1.667)	0.785
Male	141				
Female	110				
pN status		1.765(1.051,2.962)	0.032	1.791(0.960,3.341)	0.067
N0	117				
N1-N3	134				
Grade		0.426(0.251,0.725)	0.002	0.250(0.131,0.477)	<0.001
Well/moderate	191				
Low	60				
Tumor location		0.538(0.316,0.917)	0.023	0.587(0.315,1.095)	0.094
Rectum	138				
Colon	113				
NGFR expression		0.281(0.155,0.509)	<0.001	0.105(0.040,0.274)	<0.001
Low	115				
High	136				
S100A9 expression		0.535(0.308,0.930)	0.027	0.425(0.211,0.858)	0.017
Low	107				
High	144				

CI, confidence interval; DFS, disease-free survival; HR, hazard ratio; OS, overall survival.

### NGFR Regulating the Activation and Resistance of 5-FU Led to Cell-Cycle Arrest

To determine the activation and resistance mechanism of 5-FU by which NGFR increase the chemosensitivity of CRC to 5-FU, the effect of NGFR on cell cycle distribution under 5-FU was analyzed. Compared with the control groups, the ectopic expression of NGFR led to a significant increase in the number of G1-phase cells (39.5% ± 0.6% vs. 42.1% ± 3.1%, P < 0.05, before 5-FU treatment and 37.9% ± 1.2% vs. 46.2% ± 2.9%, P < 0.01, after 5-FU treatment, **Supplementary Figure 4B**). Concomitant with this increase, there was a significant decrease in the number of cells accumulating in the S-phase (32.0% ± 1.3% vs. 29.9% ± 2.2%, P < 0.05, before 5-FU treatment and 33.6% ± 1.5% vs. 26.6% ± 2.8%, P < 0.05, after 5-FU treatment, **Supplementary Figure 4B**). To investigate the resistance mechanism and 5-FU activation of NGFR increase chemosensitivity, the relationship between NGFR and the expression of TYMS, the final target of 5-FU metabolites, as well as UMP-CMP kinase, an enzyme involved in 5-FU activation, was evaluated. Data shows that TYMS expression was suppressed while NGFR over-expression transfected CRC cells under 5-FU treatment, while UMP-CMP kinase expression was elevated while CRC cells were treated with 5-FU, and NGFR transfection can increase those elevation (**Supplementary Figure 4A**). These results indicate that NGFR increase the chemosensitivity of CRC cells to 5-FU by mediating 5-FU activation through elevating UMP-CMP kinase expression, and suppressing TYMS expression, which can induce the cell cycle arrest of CRC cells under 5-FU treatment.

### NGFR Meditated the Balance of Autophagy and Apoptosis by Disrupting the Combination of Bcl-2 and Beclin-1 Proteins

To explore the mechanism by which NGFR meditates the balance of autophagy and apoptosis induced by 5-FU treatment, we overexpressed and knocked down NGFR expression in DLD1 and SW480 cells separately following 5-FU treatment to conduct co-IP, aiming to reveal changes in the combination of endogenous Bcl-2 and Beclin-1 proteins. The results showed that overexpressed NGFR considerably suppressed the combination of Bcl-2 and Beclin-1, whereas the absence of NGFR had the opposite effect. These results indicated that NGFR inhibited the interaction between Bcl-2 and Beclin-1 (**Figure 6D**).

## Discussion

The overall rate of CRC response to systemic chemotherapeutic treatment is generally less than 20%, which is attributed to drug resistance (3). The use of 5-FU, one of the most widely used agents in cancer chemotherapy, has become an important strategy to increase the sensitization of cancer cells to drug-induced apoptosis and autophagy. In our previous study (12), methylation of NGFR was observed in a substantial number of CRC cases and was associated with tumor progression and poor prognosis, whereas patients with high NGFR expression had better outcomes after 5-FU-based chemotherapy. NGFR downregulation was linked to cell proliferation and oncogenes in human CRC cells. Because NGFR binds to diverse types of ligands in different cancers, its functions are highly dependent on the cell type. NGFR can function as an oncogene, enhancing cell proliferation and promoting cancer metastasis in thyroid carcinoma and melanoma (22–24), or as a tumor suppressor, stimulating cancer cell death in prostate, bladder, stomach, and liver cancers (10, 25–28). However, the role of NGFR in the chemotherapeutic response has rarely been investigated. Until recently, the association between NGFR and chemoresistance was reported in triple-negative breast cancer cells (29).

The aim of this study was to investigate the role of NGFR in CRC chemotherapy using 5-FU as an agent. The results showed that NGFR re-expression increased the sensitivity of CRC cells to 5-FU compared to controls, which was confirmed by plate colony formation and RTCA experiments. Furthermore, fluorescence-activated cell sorting revealed that NGFR re-expression enhanced 5-FU-induced apoptosis in CRC cells, increasing their response to chemotherapy. Moreover, western blot experiments showed that NGFR re-expression increased the cleavage of caspase-3 *via* downregulation of the antiapoptotic protein Bcl-2 in CRC cells. These results suggest that NGFR may increase the response of CRC cells to 5-FU by enhancing 5-FU-induced caspase-meditated apoptosis.

To determine the pathway that mediates these enhancing effects, we used a Human OneArray v6.1 microarray to validate the expression changes in 31,741 genes’ mRNA caused by NGFR overexpression. Of those, 118 were substantially downregulated, whereas 126 were markedly upregulated. Importantly, S100A9 was the most substantially changed gene, and S100A8 was in the top five.

Members of the S100 family usually contribute to tumor growth, metastasis, angiogenesis, and immune evasion. However, S100 proteins can also act as type-specific tumor suppressors (30–33). S100A2 functions as a tumor suppressor in oral cancer and as a tumor promoter in lung cancer (34, 35). S100A7 acts as a tumor suppressor in estrogen receptor-α–positive breast cancer (36). Even though S100A9 is usually considered a tumor promoter, evidence suggests that it can also induce p53-dependent cellular apoptosis by mediating the p53 apoptosis pathway (37). Similarly, evidence indicates that S100A8 and S100A9 can induce both apoptosis and autophagy (38). Studies have shown that apoptosis and autophagy can be induced by the same stimuli and/or in the same cell type (39, 40). For example, an interplay between apoptosis and autophagy has been reported following the activation of the death receptor–dependent extrinsic apoptotic pathway by tumor necrosis factor α (TNF-α) and TNF-related apoptosis-inducing ligand (41), as well as S100A9 (38).

Investigating whether NGFR re-expression enhances 5-FU-induced autophagy in CRC cells, we demonstrated that it increased the conversion of LC3A to LC3B under 5-FU treatment, suggesting that it enhances autophagy induced by 5-FU treatment. Beclin-1 is a crucial regulator of autophagy that directly interacts with the antiapoptotic protein Bcl-2. Evidence indicates that Bcl-2 inhibits Beclin-1-dependent autophagy (42). We examined whether the interaction between Beclin-1 and Bcl-2 is involved in 5-FU-induced autophagy. Western blot analysis revealed that NGFR overexpression led to elevated Beclin-1 and reduced Bcl-2 levels and an accumulation of LC3B after 5-FU treatment. We further found that the combination of Bcl-2 and Beclin-1 was considerably suppressed by overexpressed NGFR. It is well known that the combination of these two proteins is the key mechanism regulating the autophagy/apoptosis toggle switch, serving as the platform of cross talk in these processes (43). Our results showed that the combination of Bcl-2 and Beclin-1 was disrupted in the presence of NGFR. This suggests that NGFR probably exerts its chemosensitivity-enhancing effect through autophagy regulation by disrupting the combination of Bcl-2 and Beclin-1. Taken together, our findings indicate that NGFR overexpression enhances 5-FU-induced autophagy and apoptosis by upregulating Beclin-1 and downregulating Bcl-2, as well as disrupting their combination.

In summary, this is the first study to report that NGFR increases the sensitivity of CRC cells to 5-FU treatment and enhances 5-FU-induced apoptosis with concomitantly induced autophagy. The S100A9 and Bcl-2/Beclin-1 signaling pathways are involved in the sensitization of CRC cells to 5-FU treatment. NGFR probably exerts its chemosensitivity-enhancing effect through autophagy regulation by disrupting the combination of Bcl-2 and Beclin-1. The combination of S100A9 and NGFR is a potential therapeutic efficacy prediction strategy in human CRC.

## Data Availability Statement

The original contributions presented in the study are included in the article/**Supplementary Material**, further inquiries can be directed to the corresponding author/s.

## Ethics Statement

This animal study was reviewed and approved by Institutional Animal Care and Use Committee at the Sun yat-sen University.

## Author Contributions

HC and ZY conceived and designed the experiments. JH conducted cDNA array analysis. HC and YJ conducted cell autophagy and cell apoptosis analyses. HC and XF performed the animal experiments. HC conducted western blot. HC and CC performed the data analysis. HC and YL produced the figures. ZY reviewed the data and revised the manuscript. HC wrote the manuscript. All authors contributed to the article and approved the submitted version.

## Funding

This work was supported by grants from the National Key Clinical Discipline, the National Natural Science Foundation of China (Grant no. 81802322 & Grant no. 81772594 & Grant no. 81902949), the Science and Technology Program of Guangzhou (Grant no. 201803010095), Natural Science Foundation of Guangdong Province, China (Grant No. 2019A1515011723), Medical Scientific Research Foundation of Guangdong Province, China (Grant No. A2019483) and also supported by the Fundamental Research Funds for the Central Universities (Grant no. 19ykpy09).

## Conflict of Interest

The authors declare that the research was conducted in the absence of any commercial or financial relationships that could be constructed as a potential conflict of interest.

## Supplementary Material

Supplementary Figure 1 Histograms of the fold change between the experimental and control groups (NGFR vs. PURO). Fold change represents a logarithm of fluorescence signal intensity ratios for differentially expressed genes. Log2 ratios of ≥1.0 and ≤−1.0 indicate a twofold change. The histogram shows the fold change distribution of all probes, excluding the control and flagged probes. |Fold change| ≥1 indicates differentially expressed genes.

Supplementary Figure 2 Volcano plot of the distribution of differentially expressed genes between the experimental and control groups. The dotted red and green lines represent the cutoff, a measurement of the gene expression fold change on the X-axis versus a measure of statistical significance (−Log10 [P-value]) on the Y-axis. Differentially expressed genes are established at |Fold change| ≥1 and P < 0.05 (blue dots).

Supplementary Figure 3 Hierarchical clustering of differentially expressed genes in the experimental and control groups using a Human OneArray v6.1 microarray. A hierarchical clustering tree indicates the gene expression pattern similarities of the 244 genes in the experimental and control groups. The expression levels of the 244 genes are shown by differently colored lumps: red, high (upregulated); black, medium; green, low (downregulated). S100A4, S100A8, and S100A9 are marked in red.

Supplementary Figure 4 NGFR regulate the cell cycle of CRC cells under 5-FU treatment by suppressing TYMS expression and activating 5-FU. **(A)** TYMS expression was suppressed while NGFR over-expression transfected CRC cells under 5-FU treatment. UMP-CMP kinase expression was elevated while CRC cells were treated with 5-FU, and NGFR transfection can increase those elevation. **(B)** Compare with empty-vector transfected CRC cells, NGFR transfected CRC cells showed a significant increase in the number of G1-phase and a significant decrease in the number of cells accumulating in the S-phase stage after 5-FU treatment.
