# Supplementary figures and images for: NGFR Increases the Chemosensitivity of Colorectal Cancer Cells by Enhancing the Apoptotic and Autophagic Effects of 5-fluorouracil via the Activation of S100A9
Source: Front Oncol. 2021 Apr 30;11:652081. doi: 10.3389/fonc.2021.652081 (PMC8120287; doi:10.3389/fonc.2021.652081)

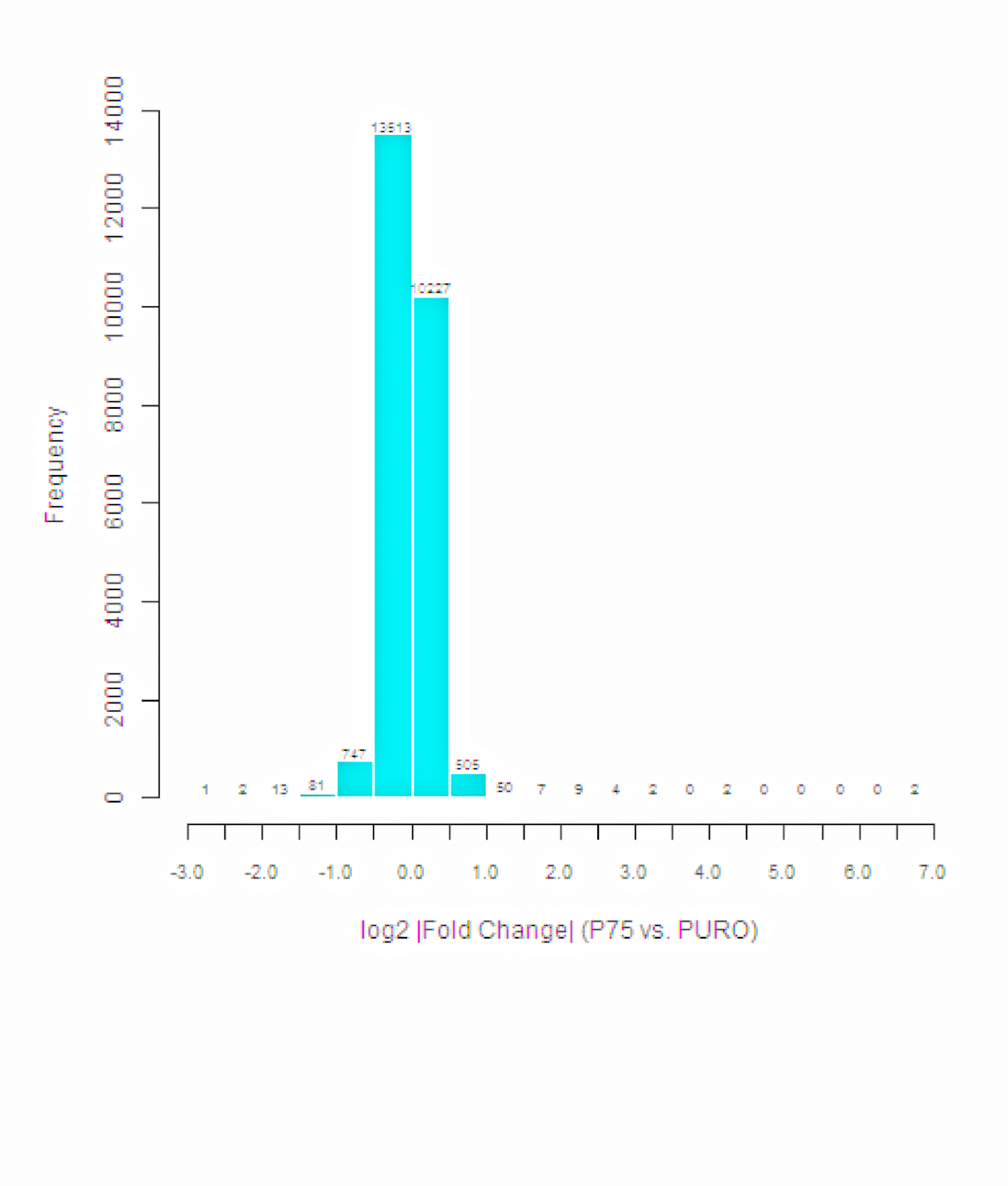

Supplement: Supplementary Figure 1 — Histograms of the fold change between the experimental and control groups (NGFR vs. PURO). Fold change represents a logarithm of fluorescence signal intensity ratios for differentially expressed genes. Log2 ratios of ≥1.0 and ≤−1.0 indicate a twofold change. The histogram shows the fold change distribution of all probes, excluding the control and flagged probes. |Fold change| ≥1 indicates differentially expressed genes. [file Image_1.tif]

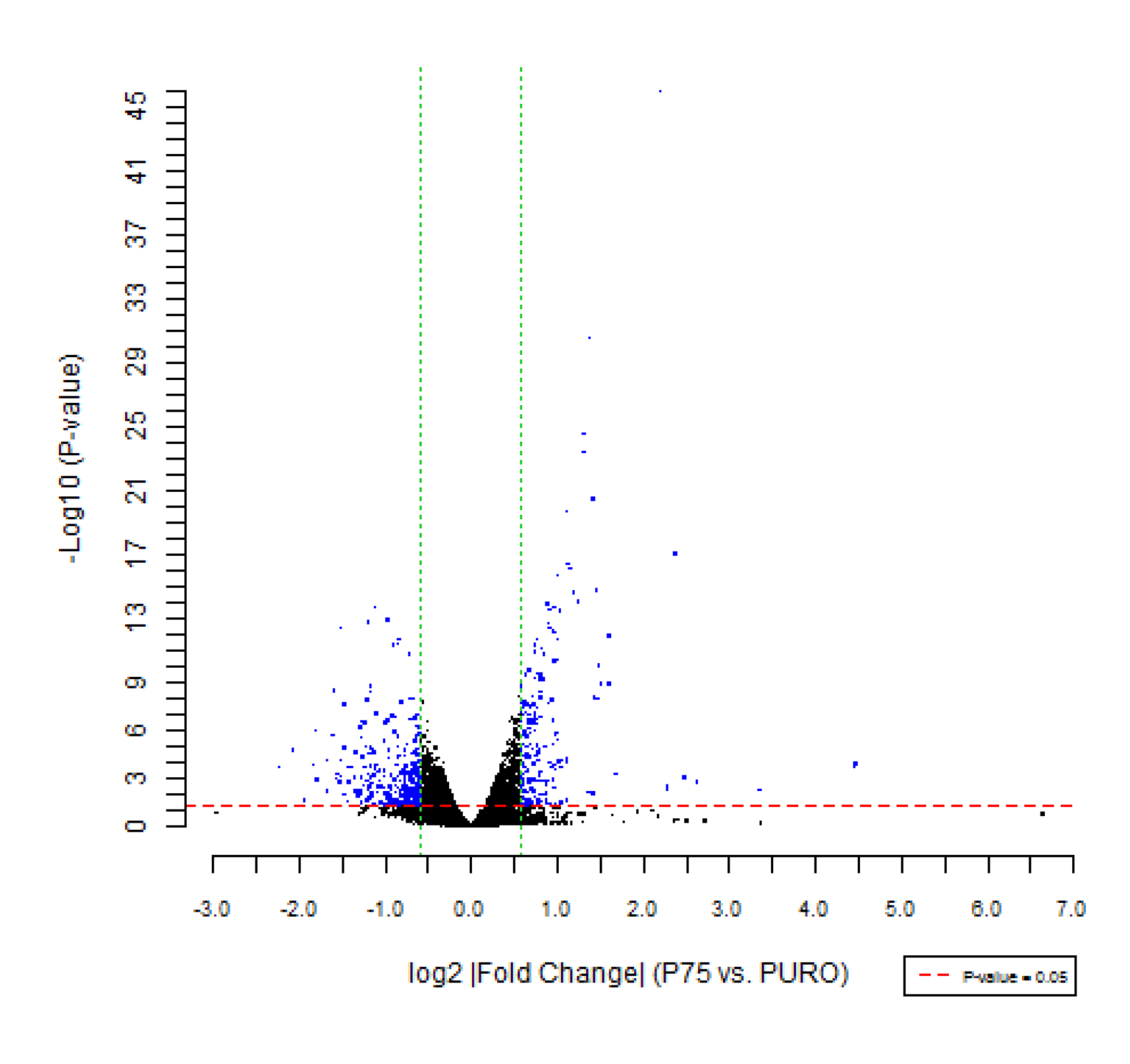

Supplement: Supplementary Figure 2 — Volcano plot of the distribution of differentially expressed genes between the experimental and control groups. The dotted red and green lines represent the cutoff, a measurement of the gene expression fold change on the X-axis versus a measure of statistical significance (−Log10 [P-value]) on the Y-axis. Differentially expressed genes are established at |Fold change| ≥1 and P < 0.05 (blue dots). [file Image_2.tif]

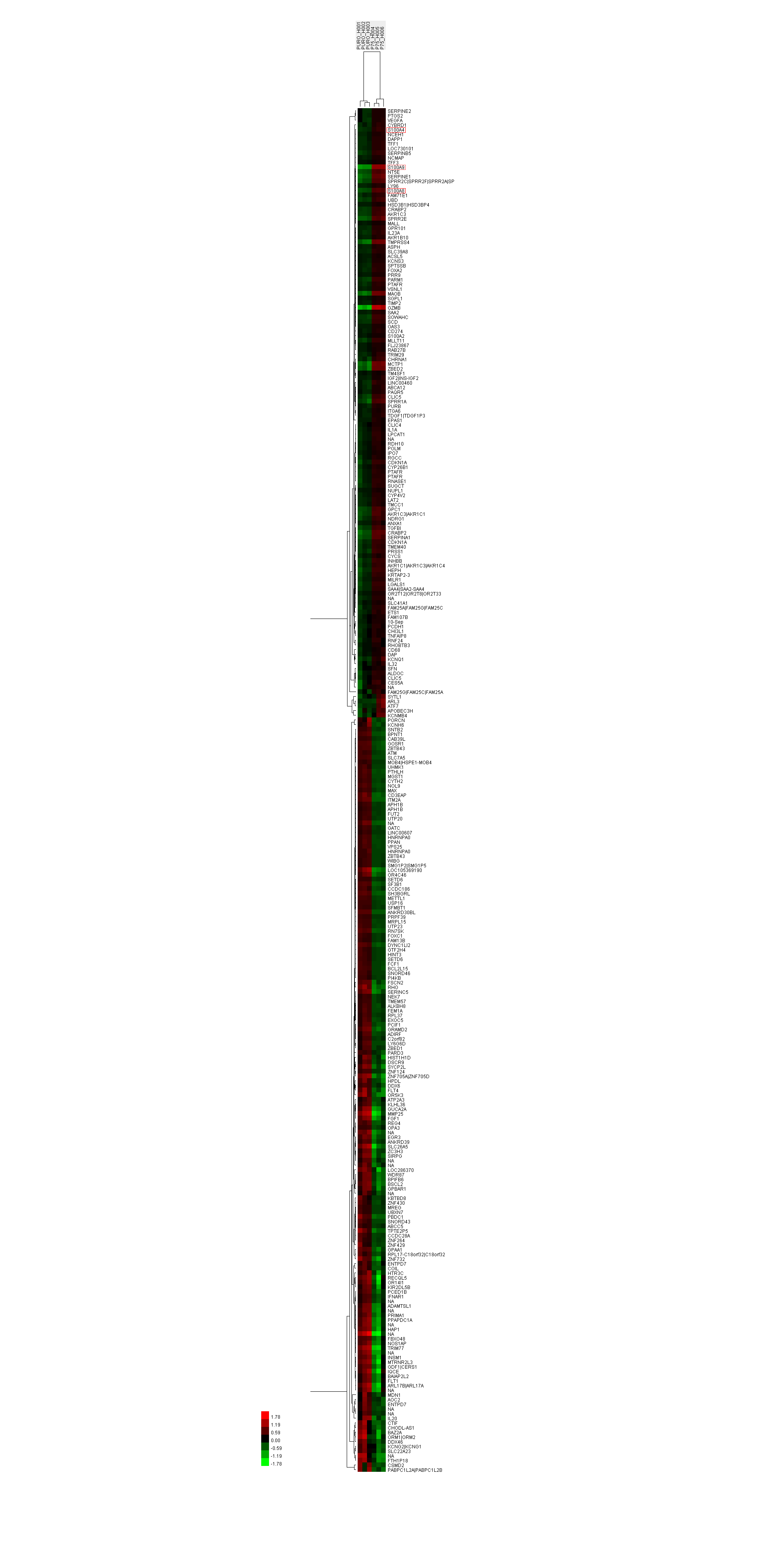

Supplement: Supplementary Figure 3 — Hierarchical clustering of differentially expressed genes in the experimental and control groups using a Human OneArray v6.1 microarray. A hierarchical clustering tree indicates the gene expression pattern similarities of the 244 genes in the experimental and control groups. The expression levels of the 244 genes are shown by differently colored lumps: red, high (upregulated); black, medium; green, low (downregulated). S100A4, S100A8, and S100A9 are marked in red. [file Image_3.tif]

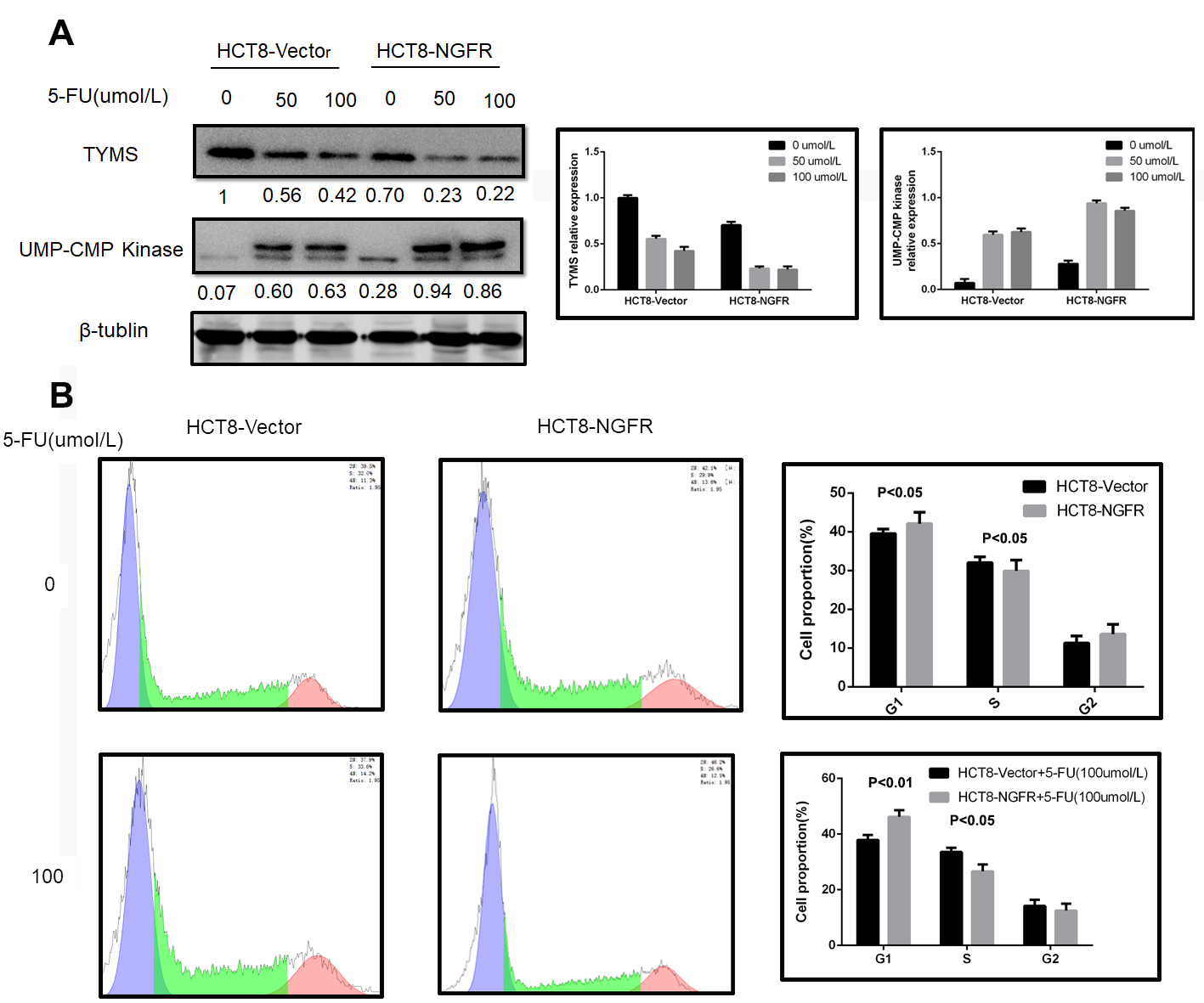

Supplement: Supplementary Figure 4 — NGFR regulate the cell cycle of CRC cells under 5-FU treatment by suppressing TYMS expression and activating 5-FU. (A) TYMS expression was suppressed while NGFR over-expression transfected CRC cells under 5-FU treatment. UMP-CMP kinase expression was elevated while CRC cells were treated with 5-FU, and NGFR transfection can increase those elevation. (B) Compare with empty-vector transfected CRC cells, NGFR transfected CRC cells showed a significant increase in the number of G1-phase and a significant decrease in the number of cells accumulating in the S-phase stage after 5-FU treatment. [file Image_4.tif]
