# Supplementary material for: NGFR Increases the Chemosensitivity of Colorectal Cancer Cells by Enhancing the Apoptotic and Autophagic Effects of 5-fluorouracil via the Activation of S100A9
Source: Front Oncol. 2021 Apr 30;11:652081. doi: 10.3389/fonc.2021.652081 (PMC8120287; doi:10.3389/fonc.2021.652081)
Supplement: Supplementary file 5 [file Table_1.docx]

Supplementary Table 1. Correlation between expression of NGFR, expression of S100A9 and clinicopathological features in 251 cases of CRC

| Factors |  | N | NGFR expression | | P-value | S100A9 expression | | P-value |
| --- | --- | --- | --- | --- | --- | --- | --- | --- |
|  |  |  | Low | High |  | Low | High |  |
| Age, years | <60 | 142 (56.6%) | 59 (51.3%) | 83 (61.0%) | 0.121 | 59 (55.1%) | 83 (57.6%) | 0.693 |
|  | ≥60 | 109 (43.4%) | 56 (48.7%) | 53 (39.0%) |  | 48 (44.9%) | 61 (42.4%) |  |
| Sex | Male | 141 (56.2%) | 66 (57.4%) | 75 (55.1%) | 0.721 | 61 (57.0%) | 80 (55.6%) | 0.818 |
|  | Female | 110 (43.8%) | 49 (42.6%) | 61 (44.9%) |  | 46 (43.0%) | 64 (44.4%) |  |
| pN status | N0 | 117 (46.6%) | 50 (43.5%) | 67 (49.3%) | 0.36 | 44 (41.1%) | 73 (50.7%) | 0.133 |
|  | N1-N3 | 134 (53.4%) | 65 (56.5%) | 69 (50.7%) |  | 63 (58.9%) | 71 (49.3%) |  |
| Grade | Well/moderate | 191 (76.1%) | 93 (80.9%) | 98 (72.1%) | 0.103 | 80 (74.8%) | 111 (77.1%) | 0.67 |
|  | Low | 60 (23.9%) | 22 (19.1%) | 38 (27.9%) |  | 27 (25.2%) | 33 (22.9%) |  |
| Tumor location | Rectum | 138 (55.0%) | 66 (57.4%) | 72 (52.9%) | 0.48 | 58 (54.2%) | 80 (55.6%) | 0.832 |
|  | Colon | 113 (45.0%) | 49 (42.6%) | 64 (47.1%) |  | 49 (45.8%) | 64 (44.4%) |  |
| Total |  | 251 (100.0%) | 115 (45.8%) | 136 (54.2%) |  | 107 (42.6%) | 144 (57.4%) |  |
