# Supplementary material for: NGFR Increases the Chemosensitivity of Colorectal Cancer Cells by Enhancing the Apoptotic and Autophagic Effects of 5-fluorouracil via the Activation of S100A9
Source: Front Oncol. 2021 Apr 30;11:652081. doi: 10.3389/fonc.2021.652081 (PMC8120287; doi:10.3389/fonc.2021.652081)
Supplement: Supplementary file 6 [file Table_2.docx]

Supplementary Table 2. Primer Sequences

| Method | Gene | Primer direction | Sequences |
| --- | --- | --- | --- |
| qRT-PCR | NGFR | Forward | 5’- GGCACCTCCAGAACAAGACCTC-3’ |
|  |  | Reverse | 5’- ACAGGGATGAGGTTGTCGGTG-3’ |
| qRT-PCR | GAPDH | Forward | 5’- TGTGGGCATCAATGGATTTGG-3’ |
|  |  | Reverse | 5’- ACACCATGTATTCCGGGTCAAT-3’ |
| qRT-PCR | S100A9 | Forward | 5’- GCAAAATGTCGCAGCTGGAA-3’ |
|  |  | Reverse | 5’- TGAACTCCTCGAAGCTCAGC-3’ |
| Vector | NGFR | Forward | 5’- CTCTAGAGATGGGGGCAGGTGC-3’ |
|  |  | Reverse | 5’- GGAATTCTCACACCGGGGAT-3’ |
